# The prevalence of self-reported diagnosed endometriosis in the Australian population: results from a nationally-representative survey

**DOI:** 10.1186/s13104-019-4114-6

**Published:** 2019-02-14

**Authors:** Rebecca Reid, Amie Steel, Jon Wardle, Erica McIntyre, Joanna Harnett, Hope Foley, Jon Adams

**Affiliations:** 1 Australian Research Centre in Complementary and Integrative Medicine, University of Technology Sydney, Ultimo, NSW 2007 Australia; 2 Endeavour College of Natural Health, Office of Research, Level 2, 269 Wickham Street, Fortitude Valley, QLD 4006 Australia; 3 Faculty of Pharmacy, The University of Sydney, Sydney, NSW 2006 Australia

**Keywords:** Endometriosis, Prevalence, Women’s health, Australia

## Abstract

**Objective:**

Currently, it is estimated that one in 10 women of reproductive age are affected by the reproductive condition known as endometriosis. However, there has been limited research and policy attention on the prevalence of endometriosis in Australia. Utilising a nationally-representative Australian sample (N = 2025), this study aimed to report on the prevalence of endometriosis in the general population and to examine the sociodemographic factors associated with the condition.

**Results:**

The results identified a prevalence rate for endometriosis of 3.4%, which aligns with previous Australian research on this topic. However, the prevalence rate from this data set is lower than the estimate prevalence from the Global Burden of Disease Study. In addition, this study reported that women self-reporting diagnosis of endometriosis, were between 40–49 years of age, with a higher proportion living in South Australia (18.2%) compared to women within the general population (8.4%). The findings highlight endometriosis as a significant health care issue warranting further research and policy attention. While acknowledging some limitations, the study provides an important foundation for further large-scale research to be conducted on this important women’s health topic.

## Introduction

Endometriosis, defined as the presence of tissue similar to endometrial lining growing outside the uterus, has recently received research attention with the investigation of new theories on the pathogenesis of the condition. These theories hypothesise that the multi-factorial disease involves interactions between epigenetics, genetics, immunology, hormonal and inflammatory aspects [1]. The disease is heterogeneous, and can cause women to experience a variety of debilitating symptomology, that negatively impacts their quality of life proportional to the severity of their symptoms [2, 3]. Such symptomology includes: dysmenorrhea; menorrhagia; pelvic pain; dyspareunia; and infertility; however, women can also be asymptomatic. Whilst research examining the implications of endometriosis has increased in recent years, a robust prevalence figure for this condition in Australia has yet to be determined. Research conducted in 1997 [4] estimated that globally one in 10 women are affected by endometriosis, with more recent research proposing up to 30% in women with infertility [5]. However, these figures are based on studies involving women undergoing laparoscopic surgery, representing a sampling bias that is non-representative of the general population. While laparoscopic surgery is the gold-standard for diagnosis, it is costly and invasive, consequently, endometriosis is commonly under diagnosed [6]. Based on these figures the World Bank in 2010 estimated that 176 million women of reproductive age worldwide are affected with endometriosis [7]. Australian longitudinal data indicates a prevalence rate of endometriosis of 3.7% within the age range of 34–39 years [8], with other estimates suggesting 560,000 women in Australia are affected [9, 10]. Whilst these figures are estimates, global data has shown a 6.4% increase in the prevalence of endometriosis between the years 1998 to 2013 [11]. However, given the difficulties in diagnosis, these figures may be inadequate and outdated.

During 2016, the Global Consortium of Investigators in Endometriosis (GCIE) published an update to the global research priorities for endometriosis [12]. Many of the GCIE priorities are of significant value to women with endometriosis. Nevertheless, without prevalence data and an understanding of the costs associated with care (including both out-of-pocket expenses and the burden on the health care system) it is difficult to justify policy change and research funding targeting endometriosis. Within the GCIE priorities, it is recommended that endometriosis organisations are established to raise public and policy-maker awareness [13]. In Australia, this movement has been establishment with endometriosis not-for-profit organisations including Endometriosis Australia [14] and EndoActive [15] as well as the Australian Coalition for Endometriosis, a peak national advocacy body representing women with endometriosis [16]. Movement to lobby the government has only just begun, and further research is needed to help alert policy-makers to this significant topic, for which prevalence data would be highly valued [17]. Whilst the above prevalence figures may be accepted globally, currently there is limited prevalence data on endometriosis in the Australian population. In direct response, this article reports the prevalence of endometriosis in Australian women from a nationally representative sample across all women of reproductive age.

## Main text

### Methods

The study employed a cross-sectional survey design utilising a purposive convenience sample of Australian adults aged over 18 years (N = 2025) who were representative of the general population with regards to age, gender and state/territory of residence [18]. Participants were recruited through a database from their membership with Qualtrics (marketing research company). Recruitment occurred between 26 July and 28 August 2017. Participation in the study required participants to provide informed consent prior to beginning the online survey. Participants, who completed the survey, received a small financial incentive for their time. The incentive is based on their membership with Qualtrics. Survey invitations were emailed to Qualtrics members who met the inclusion criteria of being 18 years or older. The survey consisted of 50 items covering demographics, health service utilisation (including complementary medicine), health status, health literacy, and health communication. Based on pilot testing, the survey completion time was approximately 15 min. The study analysed demographic data gathered from women with endometriosis as part of the larger survey. Women were included in the analysis if they selected *endometriosis* for the survey item ‘In the last 3 years, have you been diagnosed or treated for?’. This study has been reported in adherence to the STROBE Statement. Using STATA 14, a chi-squared test was used to calculate descriptive statistics and compare the characteristics of women with endometriosis to women in the general population. Cramer’s V was used to test the strength of association. A Fisher’s exact test was used to compare categorical variables. Statistical significance was set to p < 0.05.

### Results

There were 2025 respondents to the survey of which six responses were classified as incomplete or unreliable data and were removed from the data set, leaving 2019 participants. From the 2019 participants included in the survey, 652 were identified as being women of reproductive age (between 18 and 49 years). Twenty-two of these women (3.4%) reporting being diagnosed with endometriosis over the last 3 years. There was no statistically significant association between sociodemographic characteristics and women in the general population or those with endometriosis except for financial management (p = 0.021), however, this had a weak association (Cramer’s V = 0.13). Women self-reporting diagnosis of endometriosis were more likely to be within 40–49 years of age, with a higher proportion of women living in South Australia (18.2%) compared to women within the general population (8.4%). There was no statistically significant difference between women with endometriosis and women in the general population in marital status, employment, level of education, having a health care card, or private health insurance. The results are displayed in Table 1.

**Table 1:** Sociodemographic characteristics of survey participants compared with women diagnosed with endometriosis

Characteristics	Women of reproductive age (n = 630)		Diagnosed endometriosis (n = 22)		p	Cramer’s V*
	*n*	*%*	*n*	*%*		
Age (years)						
18–29	247	39.2	6	27.3	0.47	0.04
30–39	164	26.1	6	27.3		
40–49	219	34.8	10	45.4		
State						
Australian capital territory	10	1.6	0	0.0	0.61	0.07
New South Wales	191	30.3	7	31.8		
Northern territory	1	0.2	0	0.0		
Queensland	151	24.0	3	13.6		
South Australia	53	8.4	4	18.2		
Tasmania	17	2.7	1	4.5		
Victoria	141	22.4	5	22.7		
Western Australia	66	10.5	2	9.1		
Marital status						
Never married	221	35.1	9	40.9	0.52	0.07
Married	223	35.4	8	36.4		
De facto (opposite sex)	101	16.0	1	4.5		
Defector (same sex)	12	1.9	0	0.0		
Separate/divorced/widowed	73	11.6	4	18.2		
Highest qualification						
Less than year 12	66	10.5	0	0.0	0.35	0.07
Year 12 or equivalent	150	23.8	4	18.2		
Trade/apprenticeship	199	31.6	9	40.9		
University degree	215	34.1	9	40.9		
Employment status						
Full time	187	29.7	6	27.3	0.20	0.10
Part time	163	25.9	3	13.6		
Casual/temp	69	10.9	6	27.3		
Looking for work	88	14.0	2	9.1		
Not in paid work	123	19.5	5	22.7		
Financial management						
It is impossible/difficult all of the time	147	23.3	7	31.8	0.02	0.13
It is difficult some of the time	265	42.1	5	22.7		
It is not too bad	191	30.3	6	27.3		
It is easy	27	4.3	4	18.2		
Health care						
Health care card	287	45.6	12	54.5	0.26	0.03
Private health insurance	337	53.5	9	40.9	0.17	0.04

*Cramer’s V test measure of association defined as: negligible association (0.00–0.10); weak association (0.10–0.20); moderate association (0.20–0.40); relatively strong association (0.40–0.60); strong association (0.60–0.80); very strong association (0.80–1.00)

### Discussion

This is the first study that describes the prevalence of self-reported diagnosed endometriosis utilising a nationally representative sample in Australia. This data suggests that 3.4% of reproductive-aged Australian women are diagnosed with endometriosis. Based on extrapolation of the findings, this represents approximately 276,144 women in Australia [19]. This data also suggests that endometriosis affects women indiscriminately and with no socio-demographic factors influencing prevalence. Our data also represents a significantly lower number than previous estimates of 560,000 Australian women affected with endometriosis [9, 10]. The results of this study align with a recent study using the Australian Longitudinal Study on Women’s Health survey in a sample of 7427 women between 34 and 39 years of age, with a reported prevalence of 3.7% for endometriosis [8], and also align with the estimated prevalence of 4.8% between the years 2006 to 2013 by the Global Burden of Disease Study [11].

The prevalence reported in our study being lower than previous estimations, may be due to undiagnosed endometriosis and lack of policy-maker awareness which is now present with the development of the National Endometriosis Plan [20] and the Australian Coalition for Endometriosis [16]. The prevalence of endometriosis is commonly undiagnosed due to a variety of reasons including: the normalisation of abnormal menstruation by women and medical professionals [21]; lack of awareness and education of medical professionals; social concealment of menstruation [22]; and delay in diagnosis [23, 24]. These issues have been explored elsewhere and highlight women’s reports of being dismissed by medical professionals and unable to gain access for further investigation or referrals [21, 22, 25]. Without a clear understanding of endometriosis, the delay in diagnosis will continue to be hindered until women and medical professionals can clearly distinguish the condition and understand that the symptoms being experienced are not normal presentations of menstruation [26]. This is important for not only understanding the disease and clearly identifying prevalence rates, but also to acknowledge and appropriately support women suffering from this debilitating condition that has significant negative impacts on mental health and wellbeing, personal relationships, social wellbeing, sexual and physical health [3]. In addition, the asymptomatic nature of endometriosis that can occur equally impacts on diagnosis, and whilst the prevalence of asymptomatic endometriosis is not currently known, one study identified an increase in endometriosis diagnosis from women undergoing laparoscopic sterilisation [27]. Currently, laparoscopic investigation is regarded as the gold standard for diagnosis of endometriosis, with non-invasive diagnostic methods having insufficient evidence of sensitivity or specificity [10], and the invasive and resource-intensive nature of this diagnostic tool is likely to mean that many women with endometriosis will remain undiagnosed. Consequently, given that our results suggest that only half the population estimates for endometriosis in Australia have a formal diagnosis, additional research attention to non-invasive diagnostic methods for endometriosis is warranted. The over-representation of women with endometriosis in South Australia also deserves further examination of factors underlying this regional difference may offer important insights to clinicians and policy-makers. With the estimation that the prevalence of endometriosis is increasing as per the Global Burden of Disease Study [11], comes the increase in economic burden on the health care system. The economic burden of care for women with endometriosis was reported to cost $7.7 billion in Australia in 2014 [9]. In addition, women with endometriosis are reported to have a higher risk of several chronic diseases including cancers, autoimmune disease and cardiovascular disease and in Australia these diseases, are a remarkable economic burden to the health care system [28] and are identified as National Health priority areas [29]. In light of the findings of this study and given the estimated cost associated with endometriosis care, more detailed economic analyses are warranted.

### Conclusion

This study further highlights endometriosis as a significant health issue among Australian women and identifies an estimated prevalence of endometriosis that aligns with Australian longitudinal data and the 2013 Global Burden of Disease Study. Future research should focus on the challenges associated with the diagnosis of endometriosis including the development of a more efficient method for diagnosis and the relevant social implications. Due to the limitations in this study, further research is needed to clearly identify the prevalence of endometriosis in the Australian setting utilising a larger nationally representative sample including women from a larger age-bracket.

## Limitations

To the authors’ knowledge this is the first study to attempt to identify the prevalence of endometriosis in a nationally representative sample. However, the findings of this study are not without limitations. First, despite being a nationally representative sample, only a small section of this sample self-reported being diagnosed with endometriosis. Due to this, there may be under reporting of the prevalence of endometriosis in Australia from this analysis. The analysis also did not include women < 18 years and women 50 years of more. Whilst endometriosis is considered to affect women of reproductive age, the condition may still be present in women of any age. In addition, the impact of the small sample size was minimised by using Fishers exact test to examine correlations. Acknowledgment of the limitation of the sample recruitment strategy is also warranted. While the sample was drawn from participants receiving an incentive, incentives can increase the number of participants completing the survey, however this technique can cause selection bias, and both increase and decrease non-response bias [30, 31]. While this may have resulted in selection bias in the reported survey, using incentives in survey research is becoming common practise and can increase response rates and retention of participants [32]. The survey question regarding the 3 year timeframe of receiving a diagnosis of endometriosis is also a limitation as it excludes women diagnosed or being treated for endometriosis outside of this timeframe. Diagnosis of endometriosis in this survey is self-reported and does not distinguish between visual inspections via laparoscopic investigation with positive histology, the gold standard for endometriosis diagnosis [33].Consequently, this provided additional limitations regarding the objective nature of self-reporting and recall. Despite these limitations, this study represents a first national study investigating self-reported diagnosed endometriosis in Australia. Given the complexity of endometriosis, further research is needed with a larger sample of women with diagnosed endometriosis to clearly articulate the prevalence of endometriosis in the Australian community.

## Abbreviation

GCIE: Global Consortium of Investigators in Endometriosis
